# IUC26671-87 Adjuvant Pembrolizumab in RCC: Real-world experience in Northern Ireland 2022-2026

**DOI:** 10.1093/oncolo/oyag286.020

**Published:** 2026-08-21

**Authors:** S Buchanan, G Clements, C Forde, A Clayton

**Affiliations:** Northern Ireland Cancer Centre, Belfast - United Kingdom; Northern Ireland Cancer Centre, Belfast - United Kingdom; Northern Ireland Cancer Centre, Belfast - United Kingdom; Northern Ireland Cancer Centre, Belfast - United Kingdom

## Abstract

**Background:**

Adjuvant pembrolizumab has been shown to improve both overall and disease-free survival in the Keynote 564 trial for clear cell renal cell carcinoma and was approved by NICE for use in the UK in October 2022. We have investigated the real-world experience in Northern Ireland, focusing on toxicity and steroid use.

**Methods:**

A retrospective chart review of all patients who were discussed at the regional multidisciplinary team (MDT) meeting for consideration of adjuvant therapy between October 2022 and January 2026 was conducted using electronic healthcare records.

**Results:**

204 cases meeting the Keynote 564 eligibility criteria were discussed at the MDT meeting. 93 (45.5%) patients proceeded to adjuvant treatment. Pembrolizumab was prematurely discontinued in 48 (51.6%) patients – 40 (43%) due to toxicity, 6 (6.5%) due to relapse and 2 (2.1%) due to other health issues. Figure 1 depicts the range and severity of toxicities reported. Toxicity of any grade was reported by 88 (94.6%) patients. Most common toxicities included fatigue (43%), pruritus (32.2.%) and rash (28%). The most common Grade 3/4 toxicity was colitis (6.5%). Steroid treatment was required in 44 (47.3%) patients, with 29 (31.2%) patients requiring high-dose steroids (40mg prednisolone or higher). Median duration of steroid treatment was 109 days with 5 patients still on reducing steroids at the time of data collection. High-dose steroids were prescribed most frequently for colitis (11 patients) and hepatitis (9 patients). Additional immunosuppressant agents were required in 14 (15%) patients. Endocrine complications occurred in 28 patients (30.1%), 18 (19.4%) with hypothyroidism, 9 (9.7%) with hypoadrenalism and 1 (1%) with new-onset type I diabetes mellitus. There were 23 treatment-related hospital admissions with a median length of stay of 9 days. There were no treatment-related deaths.

**Conclusions:**

Pembrolizumab has been shown to improve overall survival and disease-free survival in the Keynote 564 study. Real-world data show higher rates of toxicity and steroid use than in the Keynote 564 population. Patients should be counselled appropriately to make an informed decision in the adjuvant population.

